# Antisense oligonucleotides targeting ORF1b block replication of severe acute respiratory syndrome coronavirus 2 (SARS-CoV-2)

**DOI:** 10.3389/fmicb.2022.915202

**Published:** 2022-10-26

**Authors:** Sophie Dhorne-Pollet, Christopher Fitzpatrick, Bruno Da Costa, Clara Bourgon, Jean-François Eléouët, Nicolas Meunier, Verónica A. Burzio, Bernard Delmas, Eric Barrey

**Affiliations:** ^1^INRAE, AgroParisTech, GABI, Université Paris-Saclay, Jouy-en-Josas, France; ^2^Universidad Andrés Bello, Santiago, Chile; ^3^INRAE, UMR VIM, Université Paris-Saclay, Jouy-en-Josas, France; ^4^Centro Científico y Tecnológico de Excelencia Ciencia, Vida/Andes Biotechnologies SpA, Santiago, Chile

**Keywords:** SARS-CoV-2 antisense therapy RNA virus, coronavirus, SARS-CoV-2, oligonucleotide antisense therapy, RNA therapy, ASO, RNA virus

## Abstract

The ongoing COVID-19 pandemic continues to pose a need for new and efficient therapeutic strategies. We explored antisense therapy using oligonucleotides targeting the severe acute respiratory syndrome coronavirus (SARS-CoV-2) genome. We predicted *in silico* four antisense oligonucleotides (ASO gapmers with 100% PTO linkages and LNA modifications at their 5′ and 3′ends) targeting viral regions ORF1a, ORF1b, N and the 5′UTR of the SARS-CoV-2 genome. Efficiency of ASOs was tested by transfection in human ACE2-expressing HEK-293T cells and monkey VeroE6/TMPRSS2 cells infected with SARS-CoV-2. The ORF1b-targeting ASO was the most efficient, with a 71% reduction in the number of viral genome copies. N- and 5′UTR-targeting ASOs also significantly reduced viral replication by 55 and 63%, respectively, compared to non-related control ASO (ASO-C). Viral titration revealed a significant decrease in SARS-CoV-2 multiplication both in culture media and in cells. These results show that anti-ORF1b ASO can specifically reduce SARS-CoV-2 genome replication *in vitro* in two different cell infection models. The present study presents proof-of concept of antisense oligonucleotide technology as a promising therapeutic strategy for COVID-19.

## Introduction

Coronaviruses are zoonotic pathogens that are known to infect various mammalian and avian species and to occasionally jump and adapt to new hosts. Three coronaviruses have crossed the species barrier to cause deadly pneumonia in humans since the beginning of the twenty first century: Severe acute respiratory syndrome coronavirus (SARS-CoV), Middle-East respiratory syndrome-related coronavirus (MERS-CoV) and the COVID-19-causing SARS-CoV-2 (Walls et al., 2020 and references therein). The first SARS and MERS epidemics were rapidly contained, in contrast to SARS-CoV-2, which emerged from Wuhan (China) in 2019 and reached pandemic proportions with over 600 million infections and 6.5 million deaths as of September 2022, in less than 3 years (Coronavirus Resource Center, 2022).

SARS-CoV-2 vaccines have been developed and produced at record speed (Wang et al., 2020) and around 68% of the world population is at least partially vaccinated as of September, 2022 (ourworldindata.org). The speedy development of a wide plethora of vaccine modalities has seen the rise of RNA vaccines, which can be quickly adapted to emerging variants (Topol, 2021).

As for treatment of severe cases of COVID-19, numerous drug repurposing clinical trials have been launched around the world to treat SARS-CoV-2-infected patients. New specific drugs have been tested and are now approved as more effective treatments for COVID-19 (Gavriatopoulou et al., 2021; Izda et al., 2021). Among these are several monoclonal antibodies (Roche, GSK, Lilly) and, more recently, anti-viral drugs targeting early infection steps, such as Paxolivid^®^ (Pfizer), Molnupiravir^®^ (Merck USA) and Remdesivir^®^ (Gilead). In addition, anti-inflammatory drugs such as systemic corticoids (Dexamethasone) have been recommended to treat the “cytokine storm” phase of the disease.

**FIGURE 1:**
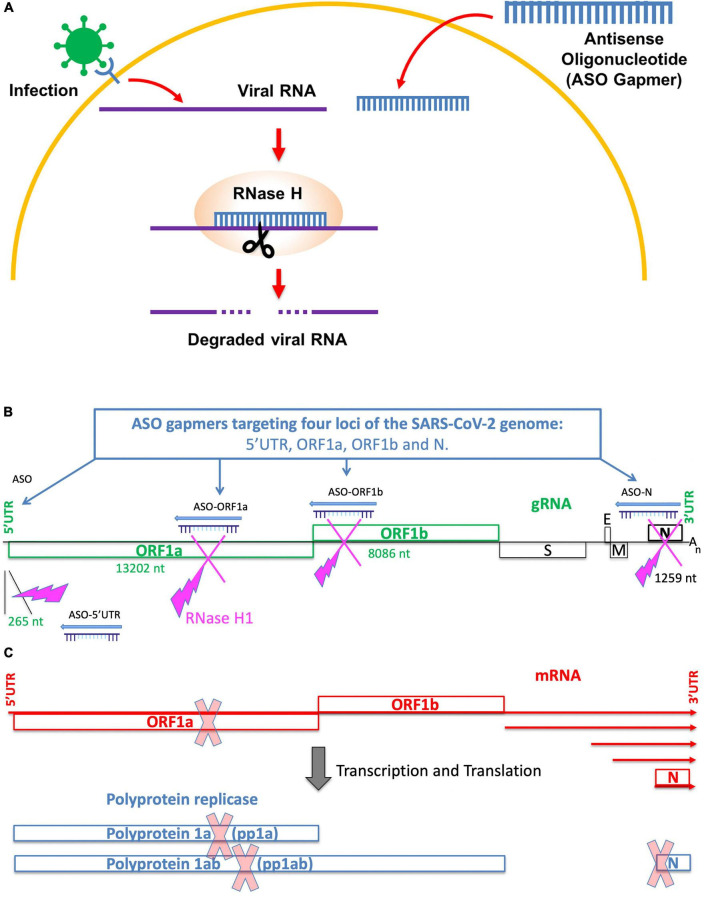
Antisense oligonucleotide therapy for SARS-CoV-2. **(A)** Principle of antisense oligonucleotide activity for silencing viral gRNA targets. ASO gapmer enters the cell where it binds to a complementary viral RNA target, which is then cleaved by cellular RNase H. **(B)** Schematic showing the concept of antisense therapy applied to transcription and replication blockage of the SARS-CoV-2 RNA genome. Genomic sequences and ORFs associated to replication and transcription are indicated in green. The length in nucleotides of each ASO-targeted region is indicated. **(C)** Schematic showing the viral gRNA and sgRNAs (in red) that share identical 3′ sequences and form a 3′ nested set of RNAs. The SARS-CoV-2 genome is a positive-sense 29.9-kb RNA molecule (gRNA) that includes a 265-nt-long 5′-untranslated region (5′UTR), followed by 22 kb (ORF1ab) encoding the replication and transcription machineries. The nucleoprotein (N) gene is located at the 3′-end of the genome just before a short 3′UTR sequence. The ORF1ab transcripts are translated into two large replicase polyproteins that are encoded by ORF1a and ORF1b, the latter being translated through ribosomal frameshift. Polyproteins 1a and 1ab are processed and their products constitute the viral replicase and transcriptase machineries, respectively. The N nucleoprotein is encoded by the smallest sgRNA.

To complete the therapeutic arsenal still required for treatment of severe cases of COVID-19, antisense therapy arises as a promising strategy. This technology is based on the use of chemically modified antisense oligonucleotides (ASOs) targeted to mRNAs, small RNAs or long non-coding RNAs for the development of treatments for different pathologies such as cancer (e.g., Vidaurre et al., 2014; Fitzpatrick et al., 2019). ASOs enter cells *in vivo* by as yet unclear mechanisms (Lobos-González et al., 2016), bind to their target RNA and the RNA in the resulting double-stranded ASO:RNA hetero-duplex is cleaved by cellular RNAse H1 (Wheeler et al., 2012; Figure 1A). Antisense therapy is a translational research topic in medicine and currently the focus of many clinical trials in oncology, myopathies, Huntington’s and many other diseases (Crooke et al., 2018). For example, a phase 1a clinical trial has been completed for the treatment of solid cancers by an antisense oligonucleotide targeted to the mitochondrial-encoded lncRNA ASncmtRNA and a phase 1b clinical trial is currently under way (ClinicalTrials.gov ID#: NCT02508441, 2015–2018). This and many other clinical trials have demonstrated that the concept of antisense therapy is coming of age and will be further developed in the near future due to its low toxicity, high specificity and low production costs.

The interest of RNA silencing has been previously established *in vitro* to curb the replication of highly pathogenic RNA viruses such as Ebola (reviewed in Kim et al., 1998; Grillone and Henry, 2008; Spurgers et al., 2008; Li et al., 2015; Tarn et al., 2021). Thus, considering the extensive body of evidence, antisense RNA therapy could be applied as an anti-viral drug by binding to and cleaving SARS-CoV-2 genomic RNA (gRNA; a positive-sense 29.9-kb RNA molecule). In the past 2 years, several studies have investigated the potential of ASOs in restraining SARS-CoV-2 replication. The 5′-, 3′-UTR and spike (S) regions have often been chosen to test the binding of ASO candidates, with different chemistries, to the primary RNA sequence (Lulla et al., 2021; Rosenke et al., 2021; Vora et al., 2022; Zhu et al., 2022; for a review, see Hegde et al., 2021).

According to epidemiological monitoring of SARS-CoV-2 variants of concern (VOC), many mutations occur along the viral genome, especially in the spike region. Thus, ASO target regions could change, resulting in mismatches between the sequences, thereby decreasing ASO efficiency in some VOCs. Consequently, our aim in the present study was to target well-conserved regions of the SARS-CoV-2 genome, avoiding target sequences under too much selective pressure such as the spike region (Weber et al., 2021). To this end, we designed ASOs targeting the 5′-UTR and the N-3′UTR regions (more conserved), as well as ORF1ab encoding polypeptides involved in transcription and replication functions at early steps post-infection (V’kovski et al., 2020). Thus, we designed *in silico* ASO gapmer candidates targeting the 5′UTR, the N-3′UTR and ORF1ab, which encodes the viral RNA synthesis machinery. The four best predicted ASO gapmer candidates were tested experimentally by different methods in human and monkey cell infection models, where we found that the ASO targeting ORF1b was the most efficient.

## Results

### Antisense oligonucleotides gapmer design and selection

We designed ASO gapmers targeting viral gRNA and the corresponding positive-sense subgenomic RNAs (sgRNAs) (Figures 1A–C). The design pipeline contemplated three complementary strategies as described in section “Materials and methods”: (i) with the use of a specific algorithm (LNA GapmeR algorithm^®^), we designed ASO Gapmers with 100% phosphorothioate (PTO) backbones and locked nucleic acids (LNA) modifications on the 5′ and 3′ ends to enhance their stability (Supplementary Figure 1); (ii) we verified that each predicted ASO targets a unique sequence, fully conserved among SARS-CoV-2 variants using the NCBI database; and (iii) we checked the predicted gRNA secondary structure around the ASO binding sites in order to determine the accessibility of each ASO to its gRNA target. We predicted 10 ASO candidates with different properties, all qualified by the algorithm as « Excellent design » (both good score and no off-targets) (Supplementary Table 1 and Supplementary Figure 2). No significant off-targets in the human genome were found for these ASO candidates, due to the high specificity of the sequence design (Supplementary Table 1). In order to better evaluate the structural constraints associated to ASO targeting, we performed a computation of the secondary structures of viral gRNA surrounding the binding sites of the ASOs. For the 5′ and 3′UTR segments, we used the MFold tool (Zuker, 2003), which allowed us to identify the positions where the viral gRNA is more accessible for ASO binding (Supplementary Figures 1–6). Finally, according to our evaluation pipeline, we selected the best four ASO gapmer candidates for further analyses to target the 5′UTR, ORF1a, ORF1b and the N gene regions (identified by bold and * in Table 1 and Figures 1B,C). The best binding site conformations, corresponding to the lower secondary structure stability of the viral RNA target sites, were found for ASO-ORF1b (Supplementary Figure 3) and ASO-N in the N-3′UTR region (Supplementary Figure 4). Their binding sites are mainly located along single-stranded loop regions, providing easy access for ASO binding. ASO-5′UTR is the only ASO predicted to target the 5′UTR and the secondary structure of its binding site lies on a 50% double-stranded RNA segment (Supplementary Figure 5). For ASO-ORF1a, the conformation of its target site along a double strand may be less accessible for binding and subsequent cleavage (Supplementary Figure 6). We also computed the ASO design on a second SARS-CoV-2 sequence (GenBank MN988668 in the NCBI database of SARS-CoV-2 genomes) and we found identical sequence conservation in the binding sites of the four selected ASO candidates ASO-5′UTR, ASO-ORF1a, ASO-ORF1b and ASO-N (Supplementary Figure 7). These ASOs were synthetized as oligonucleotides with 100% PTO internucleosidic linkages and locked nucleic acids (LNA) modifications on their 5′ and 3′ ends for *in vitro* testing (Antisense LNA GapmeRs, Qiagen, 2020; for further details see Materials and Methods).

**TABLE 1:** Characteristics of the four best ASO gapmers predicted to target 5′UTR, ORF1a, ORF1b and N, which were synthetized for *in vitro* testing.

ASO-gapmer ID#	SARS-CoV-2 targets	SARS-CoV-2 BLAST (% of genomes efficiently targeted by ASO-gapmer)	Putative human transcriptomic off-targets	ASO gapmers score within each region	Length (nt)	Melting temperature (± 1 C°)	Target positions	ASO gapmer sequence with 100% PTO backbone and LNA modifications on the 5′ and 3′ ends.
GAP1* = ASO-5′UTR	5′UTR	100%	No	1	16	50	32–47	ATCGAAAGTTGGTTGG
GAP2a* = ASO-ORF1a	ORF1a	100%	No	2	16	50	7,093–7,108	GTAGGTTGCAATAGTG
GAP2b* = ASO-ORF1b	ORF1b	100%	No	2	16	50	16,364–16,379	TACGGATTAACAGACA
GAP2n* = ASO-N	N	100%	No	2	16	50	28,420–28,435	GGTGAACCAAGACGCA

These ASOs are referenced in the text as ASO-5′UTR, ASO-ORF1a, ASO-ORF1b, and ASO-N.

*Four ASO gapmers selected among all the predicted ASOs (Supplementary Table 1) and synthetized to be tested.

### Sequence conservation of antisense oligonucleotide gapmers targets in severe acute respiratory syndrome coronavirus 2 variants of concern

In order to predict the efficiency of the four selected ASO candidates on the main identified VOCs, we performed ASO sequence alignments against all the SARS-CoV-2 genomes available on the NCBI Genbank and GISAID Tracking of hCoV-19 Variants databases, using the BLAST tool (see details in Materials and Methods). Interestingly, the four ASO gapmers displayed 100% complementarity (strand ±) to all the available SARS-CoV-2 variants (Table 1). We also aligned the four ASO gapmers against the genomes of the main VOCs and, in particular, the latest most common variants detected in Europe (Omicron BA.4, BA.5) and India (Omicron BA.2.75) during the 2022 northern hemisphere summer. Again, the four ASO gapmers showed 100% complementarity (strand ±) to these two VOCs (Table 2). Altogether, this bioinformatic screening predicted the robustness of the four ASO gapmers to target the most important VOCs of SARS-CoV-2 with perfect complementarity.

**TABLE 2:** Alignment of the four selected ASO gapmers with SARS-CoV-2 variants of concern (VOCs).

ASO gapmer ID	SARS-CoV-2 targets	SARS-CoV-2 BLAST coronavirus)	Alpha	Beta	Delta	Mu	Omicron BA.2	Omicron BA.4	Omicron BA.5	Omicron BA.2.75
ASO-5′UTR	5**′**UTR	100%	100%	100%	100%	100%	100%	100%	100%	100%
ASO-ORF1a	ORF1a	100%	100%	100%	100%	100%	100%	100%	100%	100%
ASO-ORF1b	ORF1b	100%	100%	100%	100%	100%	100%	100%	100%	100%
ASO-N	N	100%	100%	100%	100%	100%	100%	100%	100%	100%

The four ASO gapmers were 100% complementary (plus/minus strand) with all VOCs according to analyses on BLAST Coronavirus and BLAST against gRNA sequences of the main VOCs (Alpha, Beta, Delta and Omicron strains).

### Experimental evaluation of antisense oligonucleotide gapmers in cellular infection models of severe acute respiratory syndrome coronavirus-2-permissive human and simian cells

Before ASO efficiency screening, we validated the use of the human HEK-293T cell line expressing the ACE2 receptor (HEK-293T/ACE2) as an infection model, with or without transfection of negative control ASO (ASO-C). We checked the effective viral production of infected-only cells and in infected and transfected cells at 1 and 25 h post-infection by RT-qPCR of the viral N gene. The number of N RNA copies at 24 h post-infection increased 84-fold in infected-only and 71-fold in infected/transfected cells, compared to 1 h post-infection (Supplementary Figure 8). These results confirmed HEK-293T/ACE2 cells as a valid SARS-CoV-2 human infection model and showed that ASO transfection, by itself, did not interfere with viral production.

**FIGURE 2:**
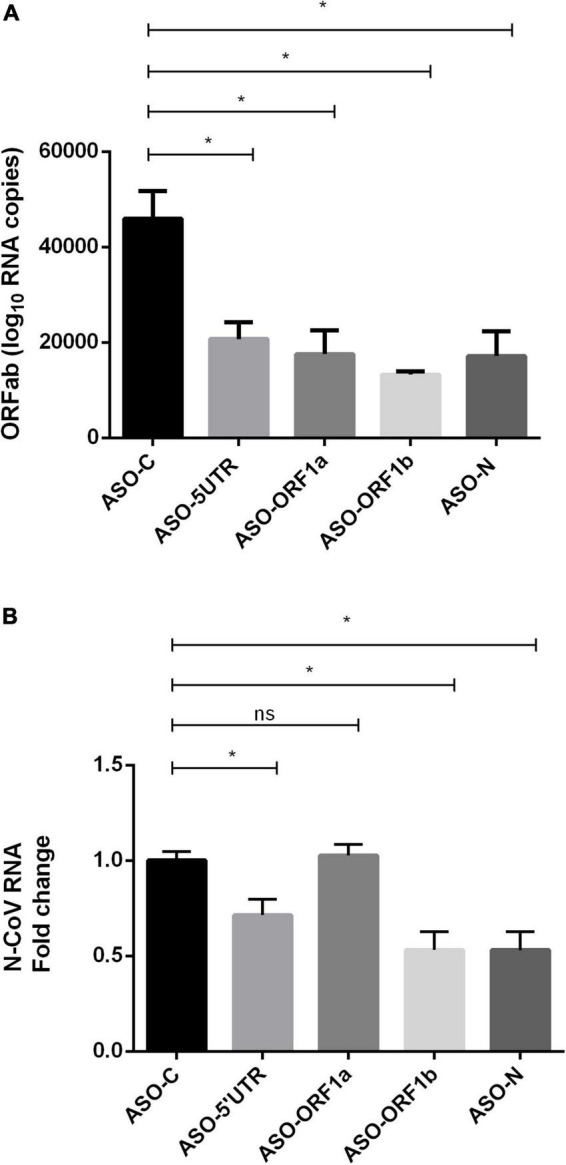
Effects of transfection of ASO gapmers on ACE2-expressing HEK293T cells infected with SARS-CoV-2. Cells were transfected with ASO gapmers, followed by SARS-CoV-2 infection 5 h later. After 24 h post-transfection, cells and supernatants were collected. **(A)** Absolute quantification of the ORF1ab copy number by RT-qPCR. All four ASO gapmers exerted a significant effect on the ORF1ab RNA copy number in comparison with ASO-C (*p* < 0.05). The reduction of the SARS-CoV-2 genome copy number reached 71% (–32,668 copies vs. ASO-C) for the treatment with ASO-ORF1b. Reduction was 55, 62, and 63% for ASO-5′UTR, ASO-ORF1a and ASO-N, respectively (*p < 0.05). **(B)** ASO efficiency measured as reduction in N-CoV expression by RT-qPCR in HEK-293T/ACE2 cells. ASO-ORF1b, ASO-N and ASO-5′UTR reduced the relative levels of the SARS-CoV-2 N transcript by 47, 47, and 29%, respectively (*N* = 5; *p < 0.05), relative to ASO-C, while ASO-ORF1a displayed no effect. Relative fold-changes to ASO-C were computed after normalization to the housekeeping genes GUSB and SDHA.

The four ASO gapmers were then evaluated in HEK-293T/ACE2 cells. Cells were first transfected with ASOs and infected with SARS-CoV-2 virus 5 h later. One day post-infection, cell culture media and cells were collected for absolute quantification of viral copy number of ORF1ab RNA by RT-qPCR. The quantity of viral genome copies significantly decreased with the four ASO treatments with –25,195 to –32,668 ORF1ab RNA copies, corresponding to a relative reduction of 55–71%, compared to ASO-C (*p* < 0.05; Figure 2A) and ASO-ORF1b transfection showed an apparently higher efficiency, with a 71% reduction. We confirmed these results by relative quantification of N-CoV RNA through RT-qPCR, where we encountered a significant 47% reduction in N-CoV fold-change in cells transfected with ASO-ORF1b and ASO-N, compared to ASO-C (*p* < 0.05; Figure 2B). ASO-5′UTR significantly reduced N-CoV fold-change by 25%, while ASO-ORF1a showed no effect (Figure 2B). To ensure that ASO-ORF1b binds and cleaves the SARS-CoV-2 gRNA, we simultaneously performed RT-qPCR on the ORF1b region using positive and negative-sense primers after ASO-ORF1b or ASO-N treatments of infected cells. As expected, we found that the viral negative-sense genomic RNA (antigenomic RNA named agRNA) was also decreased by the ASO treatment at both ORF1b and N targets (Supplementary Figure 9A), since the gRNA serves as a template for agRNA synthesis. In addition, we detected less agRNA than gRNA at ORF1b position according to RT-qPCR, as expected knowing the replication process (Supplementary Figure 9B).

**FIGURE 3:**
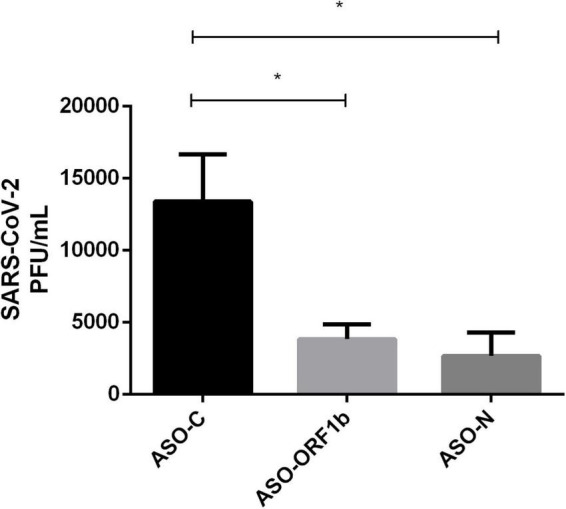
Effect of ASO transfection of infected VeroE6/TMPRSS2 cells on viral titer by PFU assay. ASO-ORF1b and ASO-N significantly reduced viral replication in infected VeroE6/TMPRSS2 cells (*p* < 0.05). In comparison to ASO-C, the reduction in viral production reached 71–80% for ASO-ORF1b and ASO-N, respectively. There was no significant difference between these two ASO gapmers. (*p < 0.05).

In order to confirm all the above results on viral replication decrease after ASO-ORF1b or ASO-N transfection, we performed a plaque forming unit assay (PFU) using VeroE6/TMPRSS2 cells to directly measure the viral titers. The PFU results demonstrated a significant reduction of viral replication of 71 and 80% for transfection with ASO-N and ASO-ORF1b, respectively in comparison to ASO-C (*p* < 0.05; Figure 3 and Supplementary Figure 10). These PFU viral titration results were consistent with the results of RT-qPCR N-CoV quantification. In addition, the use of another cell infection model demonstrated the robustness of ASO-ORF1b and ASO-N gapmer efficiency.

**FIGURE 4:**
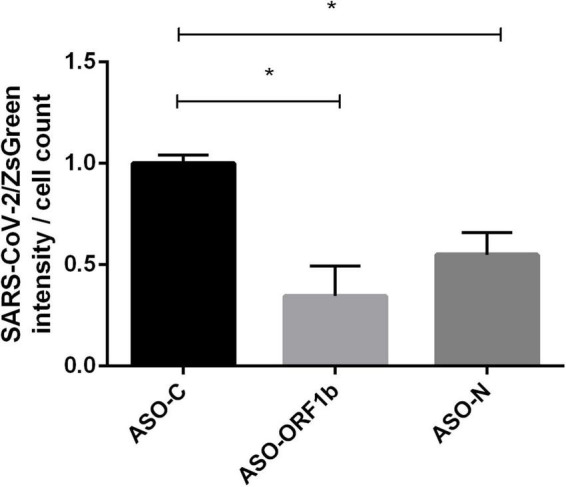
Effect of ASO transfection on VeroE6/TMPRSS2 cells infected with recombinant reporter SARS-CoV-2/ZsGreen viral particles. Cells were transfected with ASO-ORF1b, ASO-ORF-N or ASO-C and infected 5 h later with SARS-CoV-2/ZsGreen viral particles at a MOI of 0.1. Total fluorescence per well was measured by fluorometry (Tecan). Fluorescence intensity was significantly reduced in cells transfected with ASO-ORF1b (–65%) and ASO-N (–45%), compared to ASO-C (*p* < 0.05). (*p < 0.05).

To confirm these PFU results, we transfected VeroE6/TMPRSS2 cells with ASO-ORF1b, ASO-N or ASO-C, followed by infection, 5 h later, with a recombinant virus expressing a ZsGreen reporter gene (SARS-CoV-2/ZsGreen). ASO-ORF1b and ASO-N transfection of these infected cells demonstrated a significant decrease in viral protein production during replication, compared to ASO-C (*p* < 0.05; Figure 4). Interestingly, these two direct viral quantification methods are complementary and confirm the efficiency of ASO-ORF1b and ASO-N in reducing SARS-CoV-2 replication inside the cells and viral particle release into the culture media.

## Discussion

### Antisense oligonucleotides design and chemistry

By using an evaluation bioinformatic pipeline, we predicted four ASO candidates to be the most potentially efficient at knocking-down four viral regions of the SARS-CoV-2 genome: 5′UTR, ORF1a, ORF1b and the N nucleoprotein gene. The ASO gapmer chemistry, with 100% PTO linkages and nucleobase LNA modifications at the 5′ and 3′ends, provided optimal stability and specificity for binding to viral RNA targets and triggering their cleavage by the RNAse H1-mediated cellular mechanism (Figure 1; Wheeler et al., 2012). Furthermore, PTO linkages reduce melting temperature but LNA bases exert the opposite effect (Kurreck et al., 2002), thereby resulting in ASO gapmers with adequate melting temperature values. In addition, ASOs can be solubilized in PBS for cell culture transfection and are stable in RNase-free aqueous isotonic saline solution for *in vivo* injection, which is key for future therapeutic applications.

### Antisense oligonucleotides treatment efficiency

In the present study, we tested the four ASO gapmer candidates on cellular models of human and simian cells permissive to SARS-CoV-2 infection. Three ASO gapmers (ASO-ORF1b, ASO-N and ASO-5′UTR) were significantly efficient in reducing viral production in HEK-293T/ACE2 human epithelial cells. Interestingly, ASO-ORF1b displayed the highest efficiency by targeting the nsp13 open reading frame present in ORF1b and which encodes a helicase involved in viral replication. As determined in five different assays, ASO-ORF1b efficiently reduced viral replication *in vitro*: (i) it reduced the absolute number of SARS-CoV-2 genome copies during viral multiplication; (ii) it specifically reduced N-CoV transcription; (iii) it reduced the copies of ORF1b antigenomic RNAs during infection; (iv) it reduced viral production according to PFU assay; and (v) it decreased viral protein production as revealed by SARS-CoV-2/ZsGreen reporter virus. The standard N-CoV RT-qPCR quantification assay measures the copy number of the N sequence, both on SAR-CoV-2 sgRNA and gRNA (Vogels et al., 2020). Our results showed that agRNA synthesis was also affected by anti-ORF1b ASO treatment, which is unsurprising since gRNA is the template for agRNA synthesis (Masters, 2006). ASO-ORF1b treatment targets nsp3 ORF and cleaves the 30 kb-long transcript encoding pp1ab, a Zinc-binding domain-containing RNA helicase and RNA 5′-phosphatase, required for the replication process (Malone et al., 2021). In addition, the 5′ end of the ORF1b sequence contains frame-shifting elements (loop and pseudoknot) which are necessary for translating and correctly splitting the two replicase proteins pp1a and pp1ab. By cleaving ORF1b, translation of pp1ab is thus compromised (Malone et al., 2021). Altogether, we conclude that the anti-ORF1b ASO gapmer was the most efficient among our four ASO candidates for blocking viral replication in ACE-2-expressing HEK-293T cells *in vitro.*

Previous studies provided the first proof of the efficiency of ASOs in curbing replication of several viruses such as SARS-CoV. Interestingly, potent peptide-conjugated antisense morpholino oligomers (P-PMO with a phosphorodiamidate modification) are able to efficiently block SARS-CoV production in cultured cells (Neuman et al., 2005). The most effective anti-SARS-CoV antisense P-PMO that was found targeted the transcriptional regulatory sequence region present in the viral genomic 5′UTR with a viral inhibition yield exceeding 10^4^-fold. It is interesting to note that we targeted the same genomic region of SARS-CoV-2 with our ASO-5′UTR, which reduced replication in 64%. Two other studies reported the effects of antisense therapy on the reduction of SARS-CoV-2 infection *in vitro*. Rosenke et al. (2021) used polypeptide morpholinos to target the 5′UTR region of the genome on infected monkey Vero E6 cells. Four of them targeting the 5′UTR significantly reduced viral replication. Lulla et al. (2021) designed six ASO gapmers targeting the 3′UTR stem-loop 2 motif, a well-conserved region involved in transcriptional regulation. The cleavage activity of these ASO gapmers was positively tested on an astrovirus replicon model. Other trials on anti-viral activity using antisense oligonucleotide therapy have been developed with positive results in cell culture models infected with coxsackievirus, Ebola virus and HIV-1, using various types of ASO design and modifications (Jakobsen et al., 2007; Dutkiewicz et al., 2008; Chery et al., 2018).

### Perspectives of antisense oligonucleotides for antiviral therapy

For therapeutic applications, an advantage of short 15–20-mer ASO is their high ability of being physiologically absorbed into cells *in vivo* by transfection-free delivery (gymnosis) when the ASOs are administered at high dose and purity (Lobos-González et al., 2016). In general, injected ASOs display low toxicity; for example, a clinical trial in oncology demonstrated that subcutaneous injection of ASO up to 800 mg is well-tolerated and high-dose ASOs can be naturally delivered into cells (ClinicalTrials.gov ID#: NCT02508441, 2015–2018). However, in another clinical trial, reversible renal and liver toxicity was observed at a very high dose of 10 mg/kg per week during 4 weeks (Bianchini et al., 2013). Interestingly, for respiratory tract infections, ASOs can be administrated by direct intratracheal delivery through aerosol inhalation. Such aerosols can be produced by nebulization of the antisense oligonucleotide solution, as has been demonstrated in mouse models with efficient delivery to the alveolae and other pulmonary cells, and transport to the mucous layer (Karras et al., 2007; Moschos et al., 2011; Uemura et al., 2017; Brinks et al., 2019; Uemura and Kobayashi, 2019). The advantages of this inhalation delivery mode are lower costs, lower toxicity and higher local ASO concentration, readily reaching the SARS-CoV-2-infected nasal and pulmonary epithelia.

## Conclusion

We designed four ASO gapmers aimed to target and cleave selected regions of the SARS-CoV-2 genome. Three ASOs targeting the 5′UTR, ORF1b and N regions significantly reduced viral transcription and viral production in cultured cells. The ASO targeting ORF1b was the most efficient in reducing viral replication, according to all the assays that we carried out. Altogether, these *in vitro* results provide proof-of-concept of the efficiency of an anti-viral treatment based on the use of an antisense oligonucleotide targeting SARS-CoV-2 ORF1b, thereby hindering viral replication. These experimental results justify the optimization of antisense oligonucleotide therapy as a new specific COVID-19 treatment.

## Materials and methods

### Design and synthesis of antisense oligonucleotide gapmers

#### Antisense oligonucleotide gapmers design

Our strategy was based on the selection of the regions of the viral genome involved early in the replication process: 5′UTR, ORF1a and ORF1b (V’kovski et al., 2020). In addition, the 3′UTR region is interesting to target in order to block the transcription process. One additional constraint was to get an acceptable solution in the chosen region using the algorithm predicting the ASO gapmers, which was not the case for the 3′UTR. Consequently, we chose an alternative ASO in the N region, which is the most highly expressed transcript, coding for the structural proteins and therefore constituting another interesting target. The 5′UTR of the viral genome is necessary to initiate replication, while ORF1a and ORF1b code for the replicase polyproteins and were thus very interesting targets in order to block replication of the virus at early stages. For more details, the *in silico* process for design of these ASO gapmer candidates has been described in Barrey et al. (2020).

In order to maximize the probability of efficiency of our antisense oligonucleotide candidates in knocking-down the viral RNA sequences, we applied a validated method commonly used for designing ASOs used in RNA silencing. We designed 14–16-mer ASOs with 100% phosphorothioate (PTO) linkages (Supplementary Figure 1A) and locked nucleic acids (Supplementary Figure 1B) modifications at the 5′ and 3′ ends (referenced in the text as “ASO gapmers”) (Antisense LNA GapmeRs, Qiagen, 2020). These two chemical modifications are widely used in antisense oligonucleotide synthesis for stability and ultimately efficient cleavage of viral RNA targets by RNase H1 (Figure 1A). LNA modification is a bicyclic high-affinity RNA mimic with the sugar ring locked in the 3′-endo conformation (Supplementary Figure 1B). LNA nucleotides obey Watson-Crick base-pairing rules and establish stable A-helices with good base-stacking (Petersen et al., 2002). This modification increases the melting temperature Tm (+ 2–8^°^C per LNA base) and it confers nuclease resistance to oligonucleotides, increases their stability and potency in cells and, importantly, provides high specificity for binding to RNA targets (Bondensgaard et al., 2000). PTO, on the other hand, is a backbone modification in which one non-bridging oxygen atom in the α-phosphate group is replaced by sulfur (Supplementary Figure 1A), thereby conferring higher resistance to nucleases.

Design of ASO gapmers was based on the reference SARS-CoV-2 genome (NCBI Genbank Database, 2020, ID# MN908947, Wu et al., 2020; Figure 1B). ASO prediction was performed with a proprietary algorithm (LNA GapmeR^®^) on 5,000 base stretches covering the 5′UTR, ORF1a, ORF1b, and N genomic regions. For each RNA target submitted, a score was computed to sort 10 ASO candidates. After selection of the best ASOs, according to their molecular score and position on the viral genome (5′UTR, ORF1a, ORF1b, and N), we checked their specificity against the virus by searching putative off-targets with perfect alignment on the whole human transcriptome (Megablast with highly similar sequences option). The ASOs with the best scores were sorted into two categories: « Excellent design » or « Good design ». In the present study, we kept only « Excellent design » ASOs with scores ordered from 1 to 4. We computed the ASO design on each of the isolated sequences according to the annotations of the viral genome (NCBI Genbank Database, 2020, ID# MN908947); in addition, we checked the reproducibility of the results on another SARS-CoV-2 sequence (NCBI Genbank Database, 2020, ID# MN988668).

#### Analysis of the secondary structure of the severe acute respiratory syndrome coronavirus 2 ribonucleic acid genome

In order to better evaluate the suitability of ASO candidates, we performed secondary structure prediction of viral genome target segments with the MFold tool (Zuker, 2003), which can analyze up to 4000 nt-long sequences. We computed the 5′UTR region (1–265), ORF1a and ORF1b in sequences of approximately 2000 nt flanking the target sites of our ASO candidates and the last part of the N-3′UTR. The binding sites for ASO candidates were then analyzed according to the presence of single and double-stranded regions and a semi-quantitative approximation of the stability of double-stranded motifs by counting base-pairing hydrogen bonds.

#### Sequence conservation of antisense oligonucleotides targets in severe acute respiratory syndrome coronavirus 2 variants of concern

In order to predict the efficiency of the four selected ASO candidates on the main identified VOC, we performed (i) an alignment against all the SARS-CoV-2 sequences available on the NCBI database, using the Betacoronavirus BLAST tool (NCBI SARS-CoV-2 Resources, 2020, Betacoronavirius BLAST tool, accessed in April 2020 and August 2022); (ii) an alignment against the SARS-CoV-2 sequences of the main VOC identified (Wuhan, Alpha, Beta, Delta, Mu, Omicron BA.2, BA.4, BA.5, BA.2.75) available on the GISAID database, using the BLASTN tool (GISAID Database, 2022).

#### Antisense oligonucleotide gapmers synthesis

The four selected ASO gapmers and ASO negative control (ASO-C) were synthetized by Qiagen as oligonucleotides with two biochemical modifications (Antisense LNA GapmeRs, Qiagen, 2020) in order to increase their stability and specificity: 100% PTO backbone and LNA modifications on the 5′UTR and 3′UTR ends. ASO gapmers were synthesized on a 5 nmole scale and purified by desalting. Upon receipt, lyophilized ASOs were resuspended in TE (10 mM Tris, 0.1 mM EDTA) and were used at a final concentration of 100 nM.

### *In vitro* transfection and cellular infection models and antisense oligonucleotide gapmers efficiency testing

#### Cell culture

All work with infectious SARS-CoV-2 viral particles was performed in a biosafety level 3 (BSL3) facility. The SARS-CoV-2 BetaCoV/France/IDF0372/2020 strain was supplied by the National Reference Centre for Respiratory Viruses hosted by Institut Pasteur (Paris, France) and headed by Prof. Sylvie van der Werf. The human sample from which strain BetaCoV/France/IDF0372/2020 was isolated was provided by Dr. X. Lescure and Prof. Y. Yazdanpanah from the Bichat Hospital, Paris, France. BetaCoV/France/IDF0372/2020 strain was supplied through the European Virus Archive goes Global (Evag) platform, a project that has received funding from the European Union’s Horizon 2020 Research and Innovation Program under grant agreement No 653316. Vero E6 cells (African green monkey fibroblast-like kidney cells) were obtained from the American Type Culture Collection (ATCC CRL-1586; Manassas, VA) and were grown in Dulbecco’s modified Eagle’s medium (DMEM) supplemented with 10% (v/v) fetal bovine serum (FBS), 2 mM L-Glutamine, 100 U/mL Penicillin and 100 μg/mL Streptomycin. Human ACE-2-expressing HEK-293T (HEK-293T/ACE2) cells (generously provided by Mélanie Lavie, Université de Lille, CNRS, Inserm, CHU Lille, Institut Pasteur de Lille, France) were grown in DMEM supplemented with 10% (v/v) FBS, 4 mM L-Glutamine, 100 U/mL Penicillin, 100 μg/mL Streptomycin and 100 μg/mL Blasticidin (Hoffmann et al., 2020). Both cell lines were cultured at 37^°^C under a 5% CO_2_ atmosphere.

#### Severe acute respiratory syndrome coronavirus 2 amplification

Vero E6 cells were used for replication of SARS-CoV-2 BetaCoV/France/IDF0372/2020 strain. Briefly, cells were grown in T25 flasks to confluence in serum-free DMEM supplemented with 2 mM L-glutamine, without antibiotics. Cells were infected with virus at a MOI of 0.01 plaque-forming units (PFU)/cell. After 1 h of adsorption, the inoculum was discarded and 5 ml of fresh complete media was added. Cells were cultured for 3 days and observed. When cytopathic effects (CPE) became evident, supernatant was collected, cell debris was removed by centrifugation and aliquots were stored at –80°C. Viral titer was measured in supernatants by PFU assays overlaid with semisolid MEM containing 2% FBS and 2.4% Avicel (Case et al., 2020). Typical titers ranged between 10^5^ and 10^6^ PFU/mL.

#### *In vitro* transfection and cellular infection model for evaluation of antisense oligonucleotide gapmers

HEK-293T/ACE2 cells were seeded into 12-well plates, at a density of 2 × 10^5^ cells/well, in DMEM supplemented as above, but without antibiotics. After reaching 80% confluence, cells were transfected with 4 μL/well (Dharmafect1, Thermo Fisher Scientific, Waltham, MA; or Lipofectamine2000, Thermo Fisher Scientific) and 100 nM ASO gapmer. Cells were infected 5 h later with SARS-CoV-2 BetaCoV/France/IDF0372/2020 strain at MOI = 0.01 and cultured for a further 24 h. For each ASO gapmer, five biological replicates were performed.

#### Ribonucleic acid extraction

After ASO transfection and infection, cells were washed twice with PBS and total RNA was extracted using TRIzol LS Reagent (Life Technologies) and further purified using the RNeasy MinElute clean up kit (Qiagen) according to manufacturer’s recommendations. RNA yield and purity was monitored with a NanoDrop ND-1000 spectrophotometer. RNA integrity was assessed according to RNA integrity number (RIN) (Schroeder et al., 2006), on an Agilent 2100 Bioanalyzer, using the RNA 6000 nano kit (Agilent, Santa Clara, CA) following manufacturer’s directions. RIN values of extracted RNAs were between 9.3 and 10.

#### Reverse transcription

Reverse transcription (RT) was performed with the SuperScript IV first-strand synthesis system (Invitrogen). One μg total RNA and 50 ng random hexamer primers (Life Technologies) were mixed, denatured 5 min at 65°C, spun-down and stored on ice before adding the reaction mix, according to manufacturer’s instructions. RT was carried out at 23°C for 10 min and then 50°C for 45 min. The reaction was stopped by heating at 80°C for 10 min. After cDNA synthesis, RNA was degraded by incubating with 4 U RNase H for 20 min at 37°C. The reaction was stopped by heating at 70°C for 10 min and cDNAs were stored at –20°C until use. Quantity and quality of cDNA was evaluated on sixfold dilutions with the RNA 6000 Pico kit (Agilent) on an Agilent 2100 Bioanalyzer.

#### N-CoV RT-qPCR primer design

Desalted primers were obtained from Eurofins Genomics. To quantify SARS-CoV-2 gRNA copies, we used primers for the N-CoV gene designed by the Centers for Disease Control and Prevention USA (cited by Vogels et al., 2020): forward (2019-nCoV_N1-F): 5′-GACCCCAAAATCAGCGAAAT; reverse (2019-nCoV_N1-R9): 5′-TCTGGTTACTGCCAGTTGAATCTG. This set of primers have been validated for sensitivity and efficiency (Vogels et al., 2020). The N gRNA quantified by the RT-qPCR fold-change is termed “N-CoV,” according to its original name.

To quantify gRNA and agRNA in the ORF1b region, we designed specific RT and qPCR primers as follows: to amplify gRNA ORF1b region, positive RT primer (ORF1bPosRT): 5′-CTGAGGTGTGTAGGTGCCTG; forward (ORF1bPosF): 5′-GCCGCTGTTGATGCACTATG reverse (ORF1bPosR): 5′-CTCCAAGCAGGGTTACGTGT. To amplify agRNA ORF1b region, negative RT primer (ORF1bNegRT): 5′-CACGTGCTCGTGTAGAGTGT; forward (ORF1bNegF): 5′-CTGAGGTGTGTAGGTGCCTG; reverse (ORF1bNegR): 5′-TGTTCCTCGGAACTTGTCGG.

#### N-CoV RT-qPCR analysis and relative expression normalization

RT was performed as described above and qPCR was performed on a QuantStudio 12 K Flex Real-Time PCR System (Applied Biosystems) using the SYBR green PCR master mix. Amplification plots were analyzed using the QuantStudio 12 K Flex software, both for determination of Ct values and for melting curve analysis, which was applied in order to assess amplification specificity and the potential occurrence of dimers. For each primer pair, PCR efficiency was evaluated using serial cDNA dilutions. An equivalent of 500 pg of cDNA was used as template for each sample, and two technical replicates were run.

The qPCR results of target gene N-CoV were analyzed by the 2^–ΔΔCT^ method (Livak and Schmittgen, 2001) and expressed as fold-change relative to ASO-C. Ct values were normalized using two housekeeping genes, GUSB and SDHA, previously used in other studies (Panina et al., 2018; González-Bermúdez et al., 2019; Roy et al., 2020) and we checked their expression stability under all our experimental conditions. The primers for these two commonly used housekeeping genes were as follows: GUSB (Fw primer: AAGTCCTTCACCAGCAGCG; Rev primer: CCACGGTGTCAACAAGCAT); SDHA (Fw primer: TTTGATGCAGTGGTGGTAGG; Rev primer: CAGAGCAGCATTGATTCCTC). The expression stability of these housekeeping genes was computed with geNorm (M value: 0.115). Ct values of SARS-CoV-2 sequences were normalized against the geometric mean of these two housekeeping genes.

#### Validation of infection and transfection model using HEK-293T/ACE2 cells

In order to validate HEK-293T/ACE2 cells as an adequate *in vitro* infection model for SARS-CoV-2 studies (Hoffmann et al., 2020) and to discard a possible interference of lipofection, *per se*, on viral production, we infected cells with viral particles, with or without transfection of ASO-C. Cells were seeded into 12-well plates, at a density of 2 × 10^5^ cells/well. After reaching 80% confluence, half the wells were transfected with 4 μL/well Lipofectamine 2000 and 100 nM ASO-C. Four hours post transfection, all cells were infected with SARS-CoV-2 BetaCoV/France/IDF0372/2020 strain at MOI = 0.1. One hour post-infection, half of transfected cells and half of non-transfected cells were collected for RNA extraction as described above. In the remaining wells, culture media containing non-penetrated viral particles was replaced by fresh medium. At 24 h post-infection, all remaining cells were collected for RNA extraction. Viral titration was performed by RT-qPCR using a calibration curve, following the workflow described by Stephenson et al. (2021). Absolute quantification of SARS-CoV-2 genome copies was performed using a calibration relationship (Stephenson et al., 2021). Duplicate reaction mixtures included 5μL cDNA (dilution 1/50), 10 μL TaqMan master mix, and 5 μL 2019-nCoV (N protein) primer/probe mix from the TaqMan 2019-nCoV assay kit v1 (Applied Biosystems). Reaction mixtures were incubated at 95°C for 10 min, followed by 40 cycles of 95°C for 15 s and 60°C for 1 min using a StepOne real-time PCR system instrument (Applied Biosystems). All qPCRs were performed on the same run to minimize interexperimental variation. The SARS-CoV-2 copy number was estimated per 5 μL of cDNA (dilution 1/50) from a TaqMan 2019-nCoV assay control used to establish a standard curve.

#### Absolute quantification of ORF1ab ribonucleic acid copies in HEK-293T/ACE2 for antisense oligonucleotide gapmers efficiency measurements

Using the same cDNA samples analyzed by N-CoV RT-qPCR, we used the same method as above (TaqMan 2019-nCoV assay kit v1, Applied Biosystems) for absolute quantification of ORF1ab RNA copies.

### Viral titration by Plaque forming unit after transfection of antisense oligonucleotide gapmers

VeroE6/TMPRSS2 cells (Matsuyama et al., 2020) were transfected with 100 nM ASO using 3 μL Lipofectamine2000 per well in 12-well plates (Corning) and infected with SARS-CoV-2 with a MOI of 0.1 for 24 h as described above. VeroE6/TMPRSS2 cells were maintained in DMEM with 200 nM glutamine, 5% FBS and 200 μg/mL Hygromycin-B (Gibco) until seeding at 1.5 × 10^5^ cells/well. PFU assay was performed using VeroE6 cells in 12-well plates and incubated for 4 days before fixation (Case et al., 2020).

### Fluorescent quantification of VeroE6/TMPRSS2 cells infected with a recombinant reporter severe acute respiratory syndrome coronavirus 2/ZsGreen virus

*VeroE6* cells were transfected with pCC1-4K-SARS-CoV-2-ZsGreen plasmid using Lipofectamine 3000 (Invitrogen) and ZsGreen-expressing viral particles were harvested form culture media, as described by Rihn et al. (2021). VeroE6/TMPRSS2 cells were maintained in DMEM with 200 nM glutamine, 5% FBS and 200 μg/ml Hygromycin-B (Gibco) until seeding at 1.5 × 10^5^ cells/well. After 24 h, cells were transfected with ASOs as described above and infected with SARS-CoV-2/ZsGreen particles at MOI = 0.1 for 24 h. Cells were then detached with 0.05% trypsin (Gibco), and fixed in 4% formaldehyde solution for 30 min. Fluorescence was measured in a TECAN Infinite^®^ 200 PRO plate reader (Tecan Life Sciences, Männedorf, Switzerland) under 480 nm excitation and 530 nm emission in black bottom 96 well plates (Corning).

### Statistical analyses

All results are represented by average ± standard deviation (SD) of 3–5 biological replicates, unless otherwise stated. Data was analyzed using non-parametric *t*-test with Welch’s correction (GRAPHPAD PRISM6). Statistical significance was set to *p* < 0.05.

## Data availability statement

The datasets presented in this study can be found in online repositories. The names of the repository/repositories and accession number(s) can be found in the article/Supplementary material.

## Author contributions

EB, BD, and VAB developed the concept, design of ASO gapmers, and wrote the manuscript. SD-P did the experimental work with SARS-CoV-2 in the P3 facility, RT-qPCR, analyzed the data, and wrote the Materials and methods section. CF performed experimental work with SARS-CoV-2 in the P3 facility, RT-qPCR, viral titration by the PFU assay and fluorescent recombinant SARS-CoV-2 assay, analyzed the data, and wrote the Materials and methods section. BDC did the experimental work and managed the viral culture in the P3 laboratory. VAB contributed to the antisense RNA therapy strategy, secondary structure computation, and participated in writing the manuscript with her expertise in ASO therapy in oncology. J-FE contributed with his expertise in Coronavirus replication, transcription and protocol for SARS-CoV-2 infection, and including fluorescent recombinant SARS-CoV-2 assay. NM and CB contributed to the PFU viral titration method. All authors reviewed the manuscript and provided final approval for the submitted version of the manuscript.
