# Supplementary material for: Antisense oligonucleotides targeting ORF1b block replication of severe acute respiratory syndrome coronavirus 2 (SARS-CoV-2)
Source: Front Microbiol. 2022 Oct 26;13:915202. doi: 10.3389/fmicb.2022.915202 (PMC9644129; doi:10.3389/fmicb.2022.915202)
Supplement: Supplementary file 1 [file Data_Sheet_1.pdf]

Supplementary material: Figures 1-10, Tables 1-2

Figure 1: Oligonucleotide chemical modifications used in ASO gapmer synthesis to increase stability, resistance to nucleases and specificity. The two biochemichal modifications are: **A.** 100% phosphorothioate (PTO) linkages; **B.** locked nucleic acids modifications at the 5’ and 3’ ends (referenced in the text as “ASO gapmers” i.e. LNA GapmeR®, Qiagen, Hilden, Germany).

Figure 2: Relative positions of all predicted ASO candidates along the SARS-CoV-2 gRNA.

Figure 3: Partial ORF1b gRNA secondary structure centered around ASO-ORF1b (GAP2b) binding site, which is mainly on a single-stranded loop and therefore constitutes a less stable gRNA structure, which could be easily accesible for ASO binding.

Figure 4: Sequence N secondary structure, with the ASO-N (GAP2n) binding site on a double-stranded conformation.

Figure 5: Secondary structure of the 5’UTR segment; only one ASO, ASO-5’UTR (GAP1) was predicted on this region of the viral genome. ASO-5’UTR binding site presents 50% of target nucleotides in a single-stranded conformation.

Figure 6: ORF1a gRNA partial secondary structure centered around ASO-ORF1a (GAP2a) binding site, which is mainly a stable double-strand and may be poorly conformed for ASO binding.

Figure 7: Comparison of relative positions of selected ASO gapmers obtained on two different SARS-CoV-2 sequences NC_045512.2 (GenBank ID# MN908947) and GenBank ID# MN988668. Candidates are scored from 1 (best) to 10 on each fragment.

Figure 8: Control of viral proliferation at 1 and 25 hours post-infection in HEK-293T/ACE2 cells transfected or non-transfected with ASO-C. Viral production was quantified by TaqMan RT-qPCR of N-CoV using a calibration curve. At 24 hours post-infection, viral concentration increased 76 and 84-fold, compared to 1 h, in both non-transfected and ASO-C-transfected cells (**$p<0.01$, ***$p<0.001$).

Figure 9: Analysis of viral genomic (positive strand) and antigenomic (negative strand) ORF1b region. **A**. Comparison of the effect of ASO-ORF1b, ASO-N and ASO-C on antigenomic ORF1b expression (*$p<0.05$). **B.** Comparison of Ct values of viral gRNA and agRNA by RT-qPCR of positive and negative strand amplicons in the ORF1b region targeted by ASO-ORF1b (* $p<0.05$).

Figure 10: PFU assay corresponding to the results of Figure 4, performed on VeroE6/TMPRSS2 cells transfected with ASO-C, ASO-ORF1b or ASO-N before infection with SARS-CoV-2. The image shows the results of all three biological replicates performed.

Table 1: Characteristics of the ten ASO gapmer candidates predicted to target the 5'UTR, ORF1a, ORF1b and N regions of the SARS-CoV-2 genome (Genbank ID# MN908947). The ASO candidates were sorted according to their quality score within each gRNA target. All ASOs were classified as « Excellent design » by the prediction algorithm and presented no off-targets in the

human genome. The best four candidate ASOs (**bold** and marked *), ASO-5'UTR (GAP1), ASO-ORF1a (GAP2a), ASO-ORF1b (GAP2b) and ASO-N (GAP2n) were selected to be tested *in vitro* according to their properties, as described in the Results section.

Table 2: Secondary structure stability analysis of the viral gRNA targeted by the ASO gapmers. We performed an approximation by adding up the hydrogen bonds of the double-stranded segments of the target site of each ASO. The least stable structures were found on the ASO-ORF1b target site, which is the most efficient ASO.

Figure 1

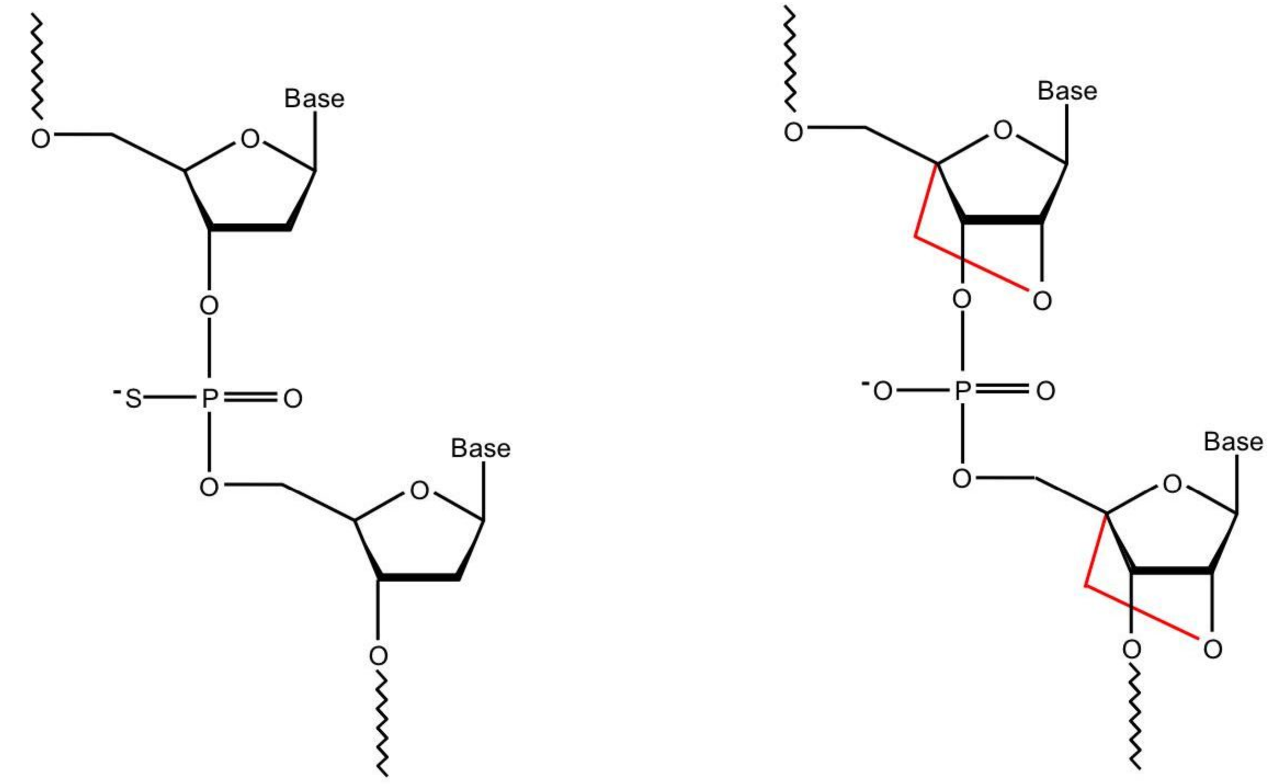

A: Phosphorothioate (PTO): a non-bridging oxigen atom is substituted by sulfur

B: Locked Nucleic Acid (LNA): sugar ring locked in the 3’-endo conformation

Figure 2

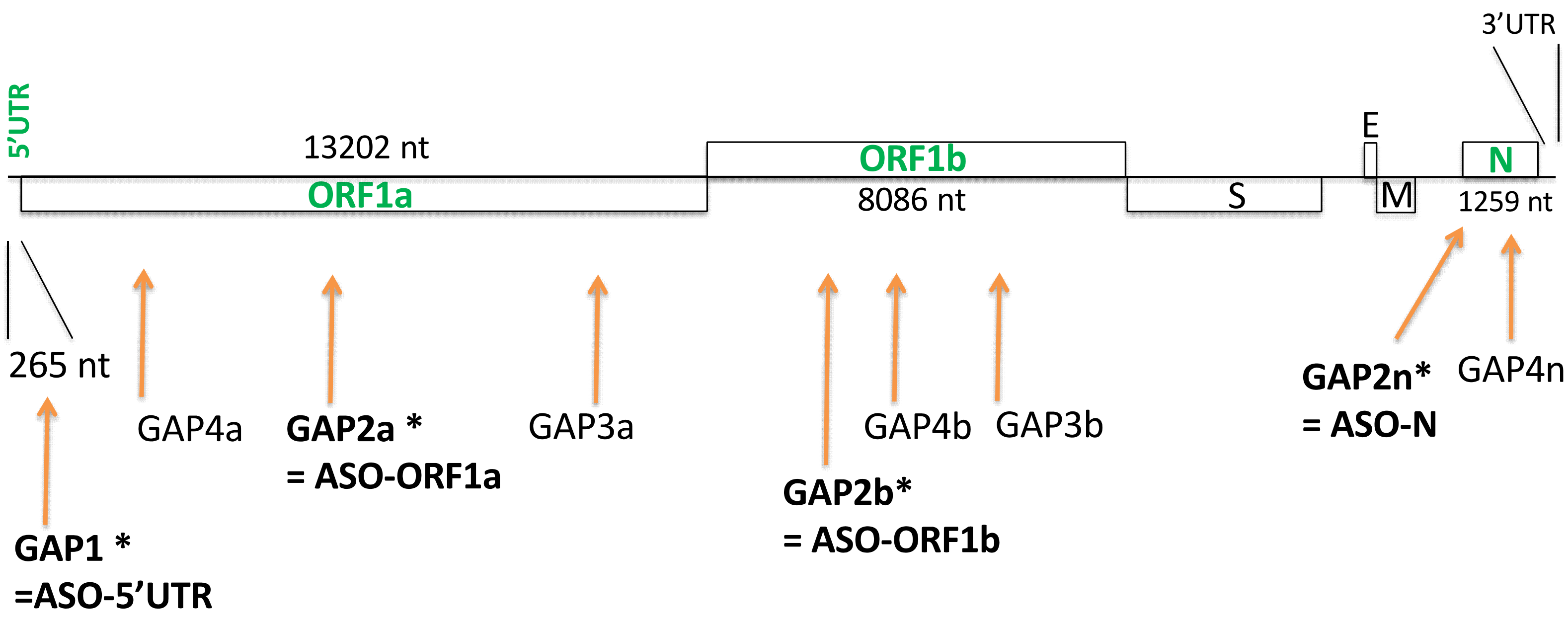
3'UTR
5'UTR
13202 nt
E
ORF1b
N
ORF1a
S
8086 nt
M
1259 nt
265 nt
GAP4a
GAP2a *
= ASO-ORF1a
GAP3a
GAP4b
GAP3b
GAP2n*
= ASO-N
GAP4n
GAP2b*
= ASO-ORF1b
GAP1 *
=ASO-5'UTR

Figure 3

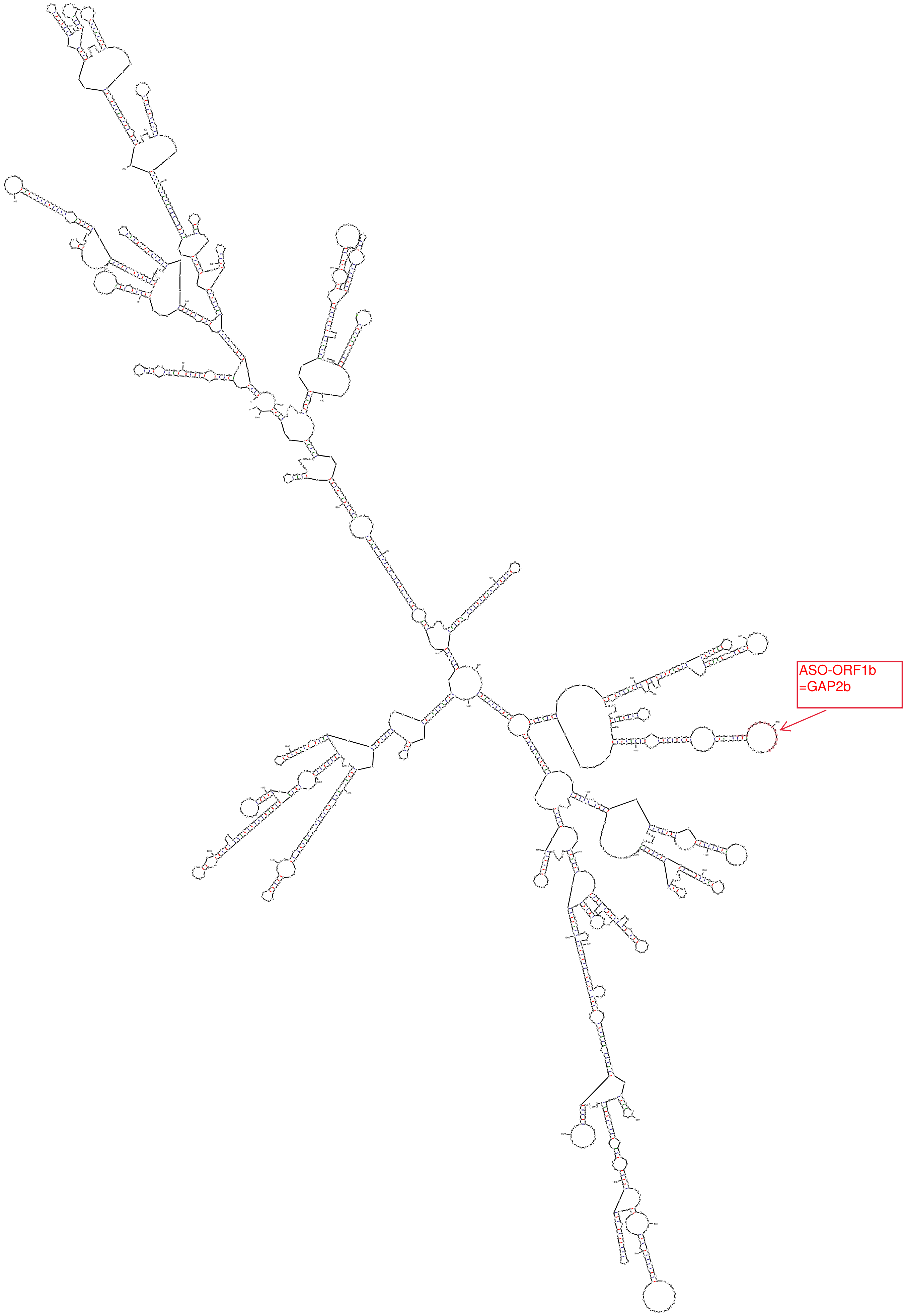

dG = -510.74 [Initially -582.00] gap2b

Figure 4

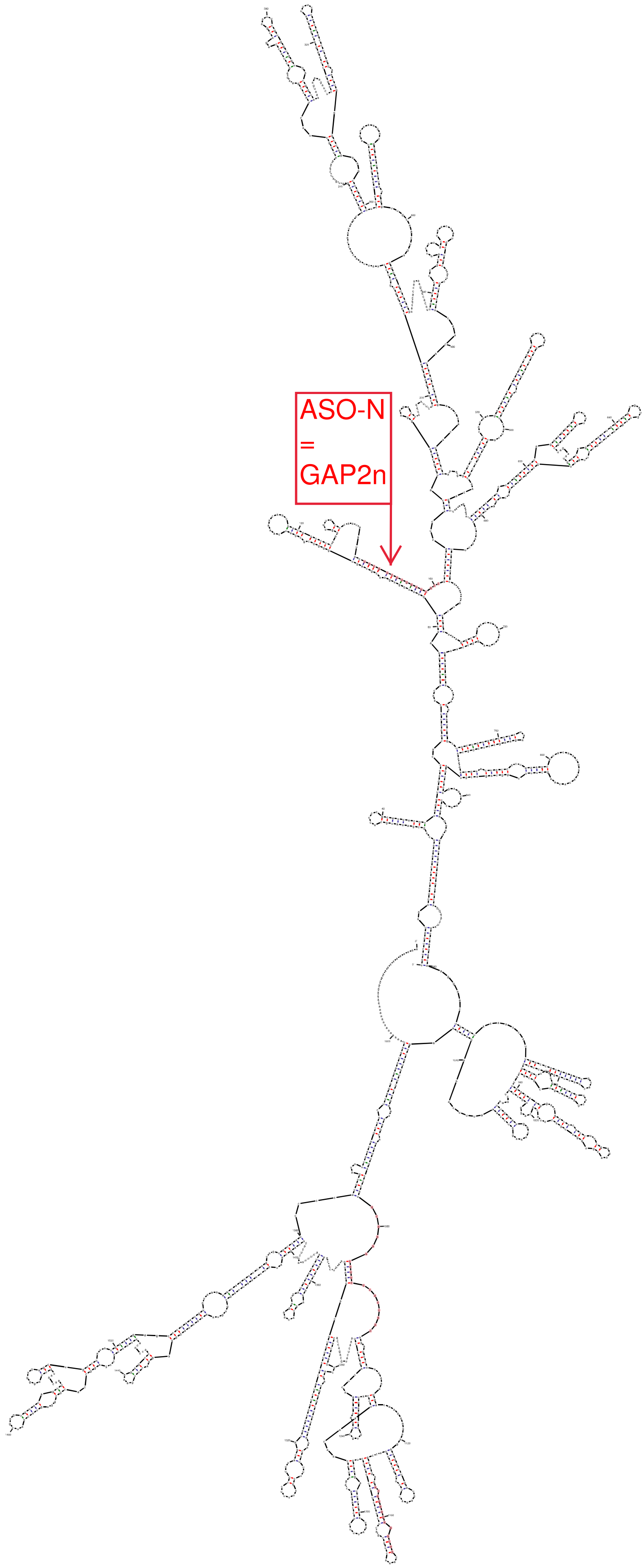

dG = -455.07 [Initially -509.50] N-3PUTR

Output of sir_graph (©)
mfold_util 4.7

Created Mon Jun 1 16:49:09 2020

Figure 5

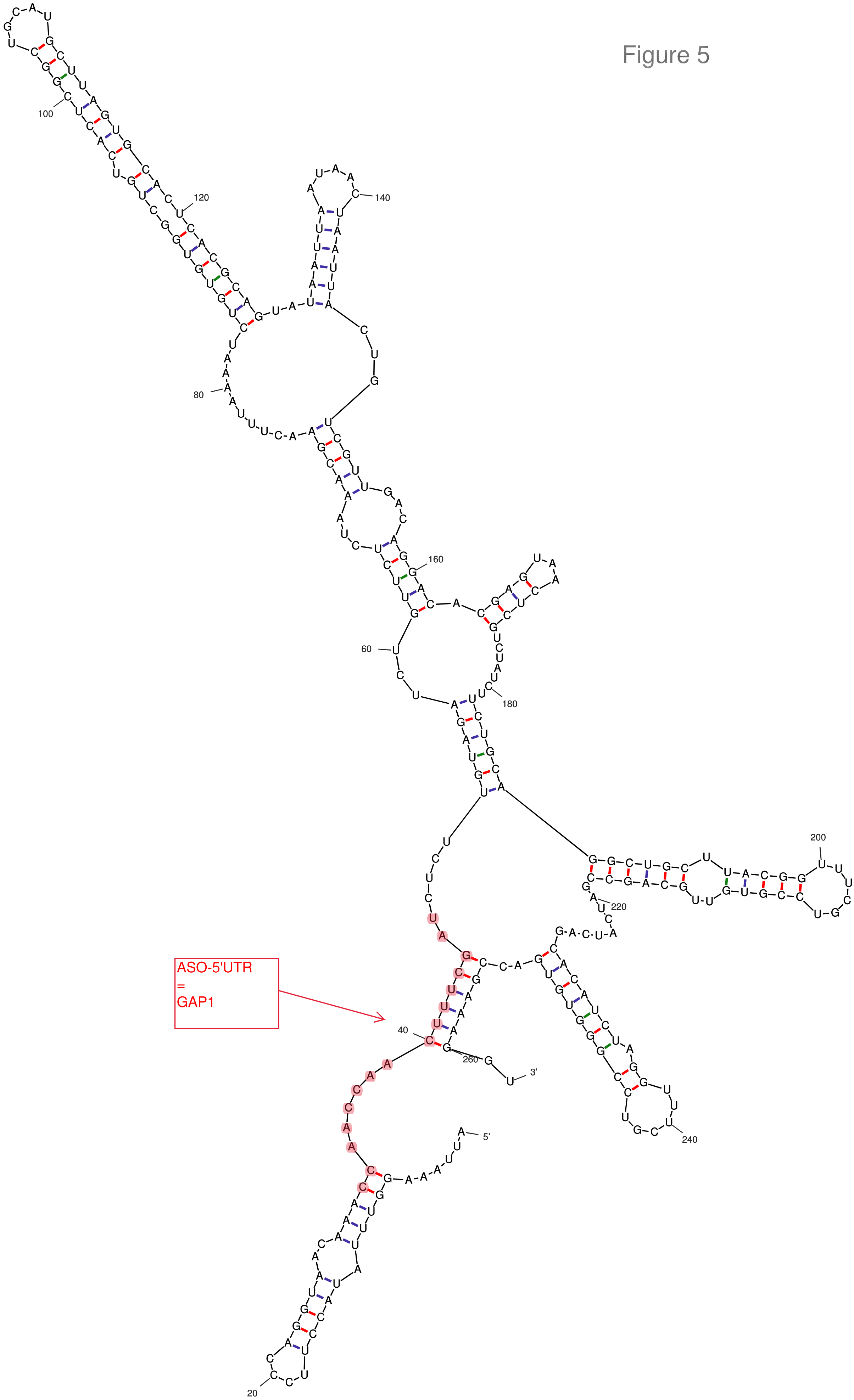

dG = -75.57 [Initially -84.10] 5UTR

Figure 6

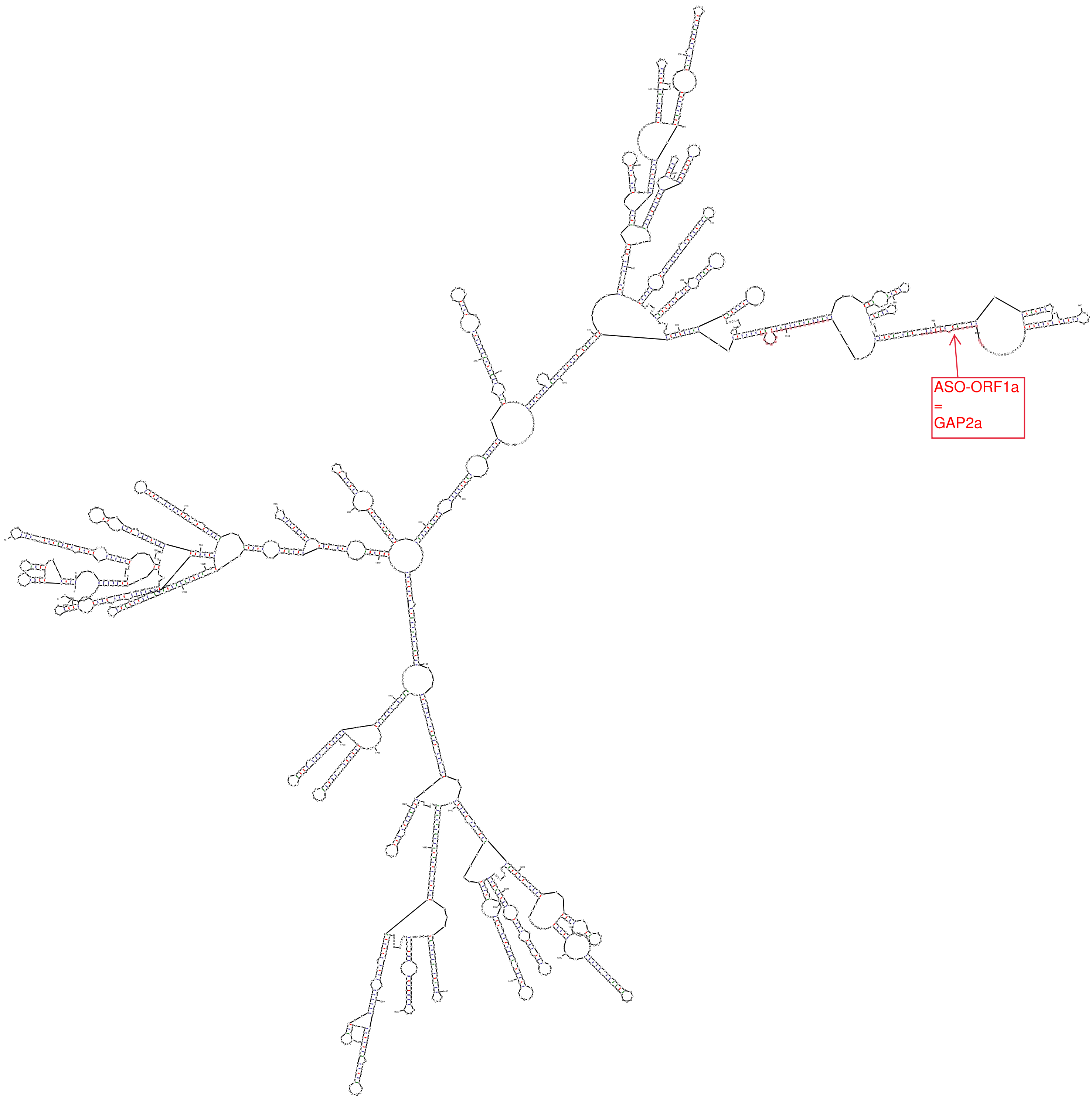

dG = -494.33 [Initially -551.00] ASO4 region

Figure 7

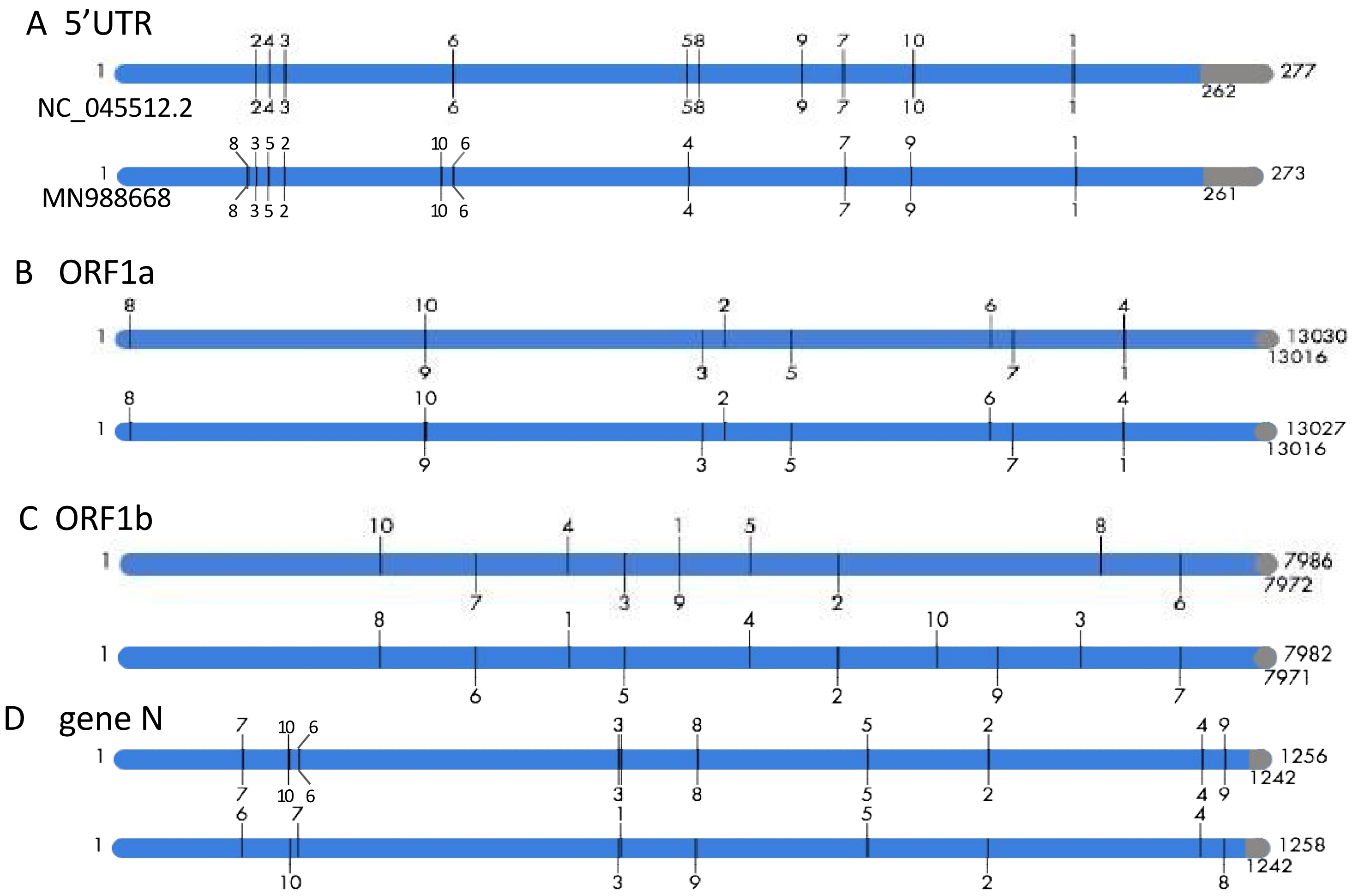
A 5'UTR
NC_045512.2
MN988668
1
277
262
273
261
B ORF1a
13030
13016
13027
13016
C ORF1b
7986
7972
7982
7971
D gene N
1256
1242
1258
1242

Figure 8

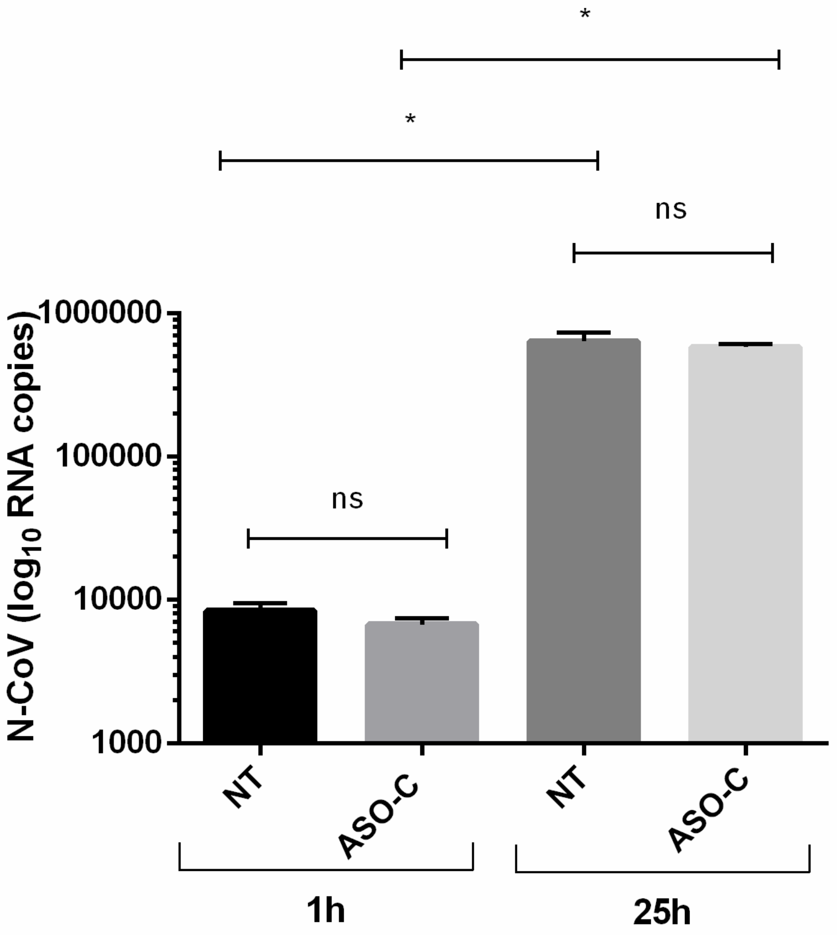
*
*
ns
ns
N-CoV (log10 RNA copies)
1000000
100000
10000
1000
NT
ASO-C
NT
ASO-C
1h
25h

Figure 9 A

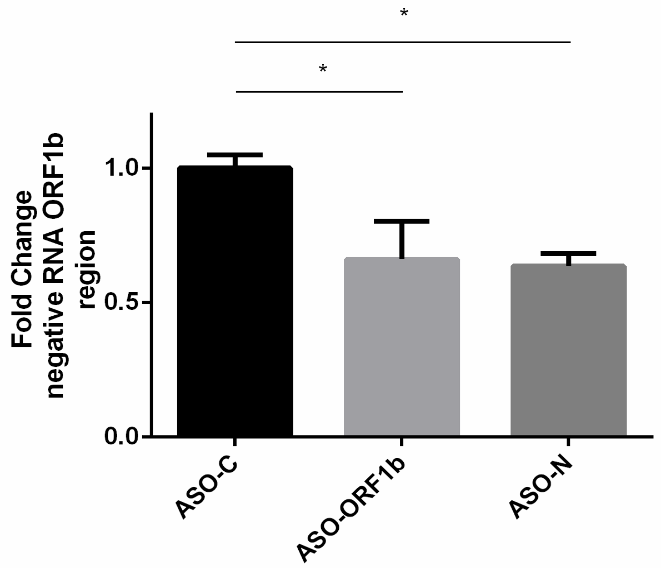

Figure 9 B

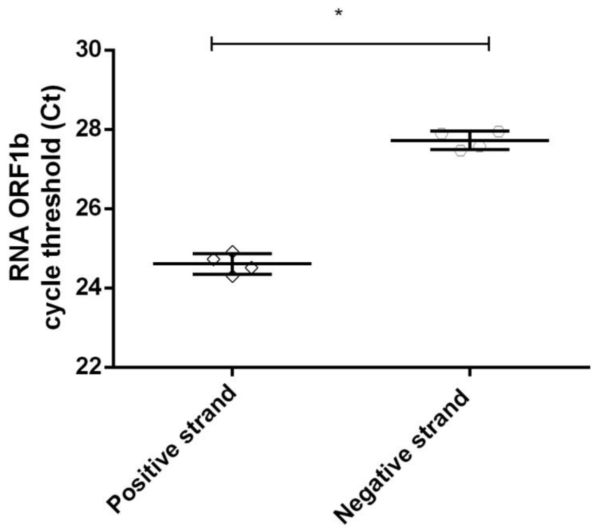

Figure 10

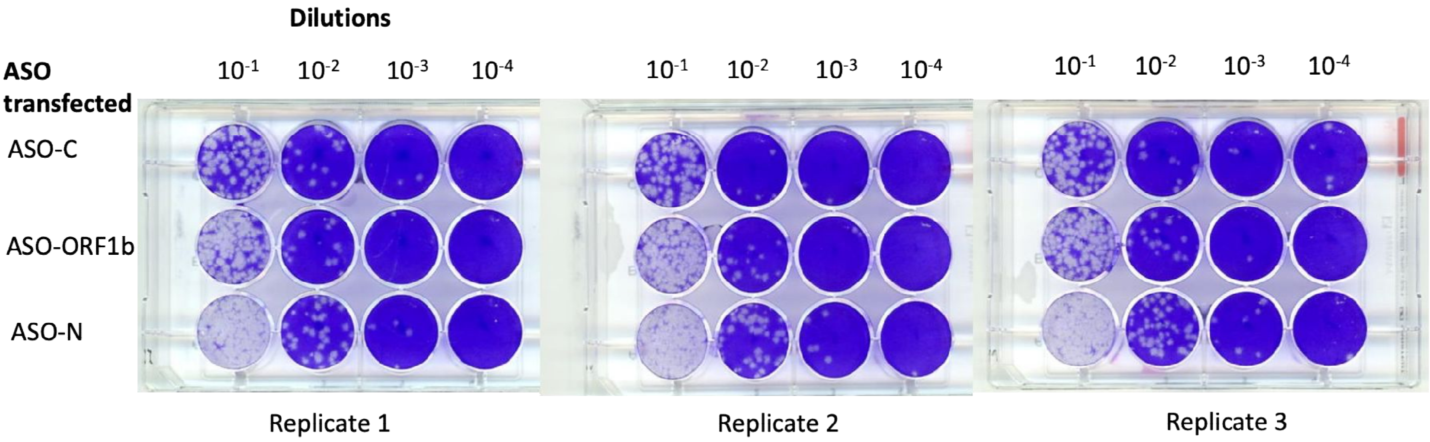

Dilutions
ASO transfected
10^{-1} 10^{-2} 10^{-3} 10^{-4}
10^{-1} 10^{-2} 10^{-3} 10^{-4}
10^{-1} 10^{-2} 10^{-3} 10^{-4}
ASO-C
ASO-ORF1b
ASO-N
Replicate 1
Replicate 2
Replicate 3

Table 1

ASO-gapmer ID#	SARS-CoV-2 targets	SARS-CoV-2 BLAST (% of genomes efficiently targeted by ASO-gapmer)	Putative human transcriptomic off-targets	ASO gapmers score within each region	Length (nt)	Melting Temperature (+/-1 C°)	Target Positions	ASO gapmer sequence with 100% PTO modification
**ASO-5'UTR = GAP1***	**5'UTR**	**100%**	**No**	**1**	**16**	**50**	**32-47**	**ATCGAAAGTTGGTTGG**
**ASO-ORF1a = GAP2a***	**ORF1a**	**100%**	**No**	**2**	**16**	**50**	**7093-7108**	**GTAGGTTGCAATAGTG**
**ASO-ORF1b = GAP2b***	**ORF1b**	**100%**	**No**	**2**	**16**	**50**	**16364-16379**	**TACGGATTAACAGACA**
**ASO-N = GAP2n***	**N**	**100%**	**No**	**2**	**16**	**50**	**28420-28435**	**GGTGAACCAAGACGCA**
GAP3a	ORF1a	100%	No	3	16	50	10133-10148	GCCAAAGACCGTTAAG
GAP3b	ORF1b	100%	No	3	16	50	18253-18268	CGCGGGTGATAAACAT
GAP4a	ORF1a	100%	No	4	16	50	3685-3700	AAGTAGAACTTCGTGC
GAP4b	ORF1b	100%	No	4	16	50	17134-17149	CTATGCGAGCAGAAGG
GAP4n	N	100%	No	4	16	50	28826-28841	TACGTGATGAGGAACG

Table 2

ASO Gapmer	SARS-CoV-2 Targets	single strand	Double strand		G:U wobble pairs	Nº hydrogen bonds
			A:U	C:G		
GAP1 *=ASO-5'UTR	5'UTR	8	3	5	0	21
GAP2a *=ASO-ORF1a	ORF1a	7	5	3	1	21
GAP2b *=ASO-ORF1b	ORF1b	12	1	1	1	7
GAP2n*= ASO-N	N	4	5	5	2	29
GAP3a	ORF1a	7	3	4	2	22
GAP3b	ORF1b	2	7	4	3	32
GAP4a	ORF1a	8	5	3	0	19
GAP4b	ORF1b	3	6	5	2	31
GAP4n	N	7	2	5	2	23
